# Substantial Overview on Mesenchymal Stem Cell Biological and Physical Properties as an Opportunity in Translational Medicine

**DOI:** 10.3390/ijms20215386

**Published:** 2019-10-29

**Authors:** Heba Abdelrazik, Emanuele Giordano, Giovanni Barbanti Brodano, Cristiana Griffoni, Elena De Falco, Alessandra Pelagalli

**Affiliations:** 1 Department of Clinical Pathology, Cairo University, Cairo 1137, Egypt; hebanabil@gmail.com; 2 Department of Diagnosis, central laboratory department, Istituto di Ricovero e Cura a Carattere Scientifico (IRCCS) Ospedale Policlinico San Martino, 16131 Genoa, Italy; 3 Department of Electrical, Electronic and Information Engineering “Guglielmo Marconi” (DEI), University of Bologna, 47522 Cesena, Italy; emanuele.giordano@unibo.it; 4 Department of Oncological and Degenerative Spine Surgery, IRCCS Istituto Ortopedico Rizzoli, 40136 Bologna, Italy; giovanni@barbantibrodano.com (G.B.B.); cgriffoni@hotmail.com (C.G.); 5 Department of Medical-Surgical Sciences and Biotechnologies, Sapienza University of Rome, 04100 Latina, Italy; 6 Mediterranea Cardiocentro, 80122 Napoli, Italy; 7 Department of Advanced Biomedical Sciences, University of Naples “Federico II”, 80131 Naples, Italy; alpelaga@unina.it; 8 Institute of Biostructures and Bioimages (IBB), National Research Council (CNR), 80131 Naples, Italy

**Keywords:** mesenchymal stem cells (MSC), translational medicine, angiogenesis, immune modulation, bone regeneration, 3D scaffolds, biomaterials

## Abstract

Mesenchymal stem cells (MSC) have piqued worldwide interest for their extensive potential to treat a large array of clinical indications, their unique and controversial immunogenic and immune modulatory properties allowing ample discussions and debates for their possible applications. Emerging data demonstrating that the interaction of biomaterials and physical cues with MSC can guide their differentiation into specific cell lineages also provide new interesting insights for further MSC manipulation in different clinical applications. Moreover, recent discoveries of some regulatory molecules and signaling pathways in MSC niche that may regulate cell fate to distinct lineage herald breakthroughs in regenerative medicine. Although the advancement and success in the MSC field had led to an enormous increase in the amount of ongoing clinical trials, we still lack defined clinical therapeutic protocols. This review will explore the exciting opportunities offered by human and animal MSC, describing relevant biological properties of these cells in the light of the novel emerging evidence mentioned above while addressing the limitations and challenges MSC are still facing.

## 1. Introduction

Thanks to more than two decades of extensive research, scientific evidence confirms a promising potential for mesenchymal stem cell (MSC)-based therapeutic strategies. This is largely due to the advancement in the knowledge of their basic biological properties, which was translated into clinical opportunities. MSC are isolated from different tissue/organs of both humans and animals. Their exclusive properties such as: (1) multipotency, (2) ability to adhere to plastic surfaces, (3) expression of specific cell surface antigens [1] and (4) potential to differentiate into osteo-, adipo- and chondrogenetic lineages [2] have engaged MSC in the regenerative medicine arena. Concerns are, however, still open about MSC-based approaches in clinics. New concepts are emerging though to help. The ability of MSC to migrate and home into injured sites in response to chemo attractants or inflammatory factors [3] offers an opportunity for their use as cellular vehicle for drugs or gene carriers [4]. Their direct interplay with the cells of the immune system opens a promising path for the clinical use of their immunomodulatory properties (indeed, being once considered as immunosuppressor cells, MSC are presently credited with a broader immunomodulation potential according to the local microenvironment). In addition to direct interaction with cells in their vicinity, active MSC-derived products, such as secreted chemokines or exosomes, are explored as substitute mediators of local MSC action [5].

Due to the variety of clinical applications that MSC maybe engaged in, this review will focus and highlight some emerging evidence about the biological and physiological properties of both human and animal MSC. Promising animal models to monitor MSC in order to improve their homing and survival in translational medicine settings will be considered. MSC interaction with different cells of the human body including immune cells for immunomodulation and endothelial cells for angiogenesis and bone healing will be covered. Their interplay with biomaterials as a therapeutic option will also be adressed. Present challenges in their translation into clinical practice will be finally highlighted.

## 2. Current Aspects of Animal-Derived MSC as Experimental Models for Therapeutic Protocols

MSC isolated from different tissue sources offer the basis for research studies focusing on their characterization to address potential therapeutic options. Animal-derived MSC provide an abundant resource to explore the specific properties of these cells to evaluate their potential functional benefits. In fact, the possibility to isolate and characterize specific biological functions of animal MSC both from adult and young tissues offers new possibilities for their therapeutic potential. Human bone marrow MSC were the first source discovered and the most frequently studied [6]. They are considered as the “gold standard” upon which other sources are compared. Although some studies evidenced that MSC obtained from human and animal species show differences in terms of their structural and biological properties, e.g., accessibility, yield, expansion and sub culturing potential, both simulate the basic characteristics of MSC; self-renewal, plasticity, multi-lineage differentiation, immune-modulation and anti-inflammatory properties. Thus, human and animal-derived MSC go side by side as supplementary and complimentary to each other [7]. In order to further explore specific mechanisms for tissue repair, several studies have been focused on the effects of different molecular and growth factors within in vivo animal models. These findings strengthen the idea that MSC could be considered factories of small growth factors able to help tissues to autoregulate the biochemical milieu in order to ensure normal physiological repair. However, more research is needed to confirm this conclusion considering variability among animal species and differences in modulate tissue responses.

Among animal species, canine MSC (cMSC) can be considered a good model to evaluate potential biological mechanisms that could be exploited for their possible application in therapy. cMSC isolated from different tissue sources (BM, adipose tissue and synovium) demonstrated similarities to human MSC (hMSC), apart from minor differences [8]. A discrete scientific interest on canine MSC has been moved to investigate molecular mechanisms exerted by several growth factors on differentiative cellular processes. In this regard, Devireddy et al., [9] demonstrated that PDGF and bFGF synergistically promoted the growth and proliferation of adipose tissue-derived cMSC while TGF-ß1, that normally facilitates hMSC proliferation, decreased cMSC population (Table 1). cMSC showed higher CFU potential vs. hMSC, in addition to the enhanced proliferation capacity of synovium and adipose tissue cMSC [8,10] suggesting that these cells could represent a promising source for canine cartilage regeneration and for their utilization as a clinical model for human medicine.

Regarding the potentialities of growth factor for MSC biology during regenerative processes, data from Mellado-López and co-workers demonstrated that plasma rich in growth factors (PRGF) from platelet-rich plasma significantly improves cell survival of ASCs when are exposed to proapoptotic concentrations of hydrogen peroxide (used for mimicking oxidative stress as occurs at site of injury and/or transplantation area) by inducing phosphorylation of AKT [11]. These findings indicate that canine and human ASCs show a comparable response to PRGF with regard to cell proliferation, cell differentiation, and AKT induction (Table 1).

More recently, other studies evidenced the role of the fibroblast growth factor-2 (FGF-2) in improving canine BM-periadipocyte cells (PAC) proliferation [17]. In the same paper, pre-treatment with FGF-2 was also able to improve chondrogenesis, affirming that it could act making spheroids impervious to the inhibitory effect of FBS during chondrogenic differentiation.

Accordingly, spheroids tend to express more cartilaginous ECM because of their oxygen gradient in their microenvironment, which exposes cells in the central area to hypoxic conditions, therefore enhancing chondrogenesis [12] (Table 1). Other finding from the same authors demonstrated that additional growth factors such as TGF-β1 and growth differentiation factor-5 (GDF-5) increased glycosaminoglycan deposition, thus increasing type II collagen expression without inducing hypertrophic differentiation [17]. These findings suggest that, when combined with TGF-β1, GDF-5 enhances in vitro articular cartilage regeneration (Table 1).

Other sources of cMSC can be the ovaries and placenta. In particular, placental cMSC demonstrated adequate paracrine secretory factors (IL-6, IL-8, MCP-1, and VEGF) which facilitated neural networks formation when co-cultured in the presence of SH-SY5Y cells. Ovary-derived cMSC showed morphological kinetic properties comparable to adipose tissue-derived cMSC. The latter demonstrated high capacity to differentiate not only into adipo-, osteo- and chondrogenic tissue, but also towards neurogenic and endodermal lineages [18].

In addition to canines, other animals can be used as sources for MSC for translational studies in regenerative medicine. Ovine species offered the setting for several preclinical studies relevant to human physiology [19,20,21]. It has been well demonstrated that ovine BM-derived aspirates showed high percentages of CFU-F cells similar to human counterparts [6,22]. However, ovine MSC (oMSC) show a reduced proliferation capacity going up to 6 passages [23,24,25], as distinct from Ad-MSC [26]. In this regard, a recent review examining the importance of oMSC for human medical applications showed differences in proliferation ability between oMSC from several tissue sources, which appears as an aspect to be considered when their clinical applications are envisaged [27]. On the other hand, Rhodes et al., concluded that age and breed variation do not significantly affect the number or the proliferation of BM-derived oMSC from both fresh and frozen cells [28].

Recently, new therapeutic approaches, such as the use of ovine peripheral blood derived MSCs (PB-MSCs) in skin lesion, were tested. Results demonstrated a discrete efficacy in PB-MSCs showing a good skin re-epithelialization. The ability of these cells to speed up the formation of granulation tissue, stimulate neovascularization, and increase structural proteins and skin adnexa, suggests their future application for deeper lesions [29].

Similarities between the human and porcine anatomy make the latter an attractive model for preclinical studies on MSC. Pig MSC (pMSC) characterized for their morphology revealed homogenous cell populations (size and granularity) coherently with flow cytometric results obtained from their hMSC counterparts [30]. Recently, a study focusing on the characterization of the optimal BM site for the isolation of pMSC showed that a different degree of starting material can be found among BM sources (sternum, humerus, tibia, femur), although they share similar phenotypes and mesodermal differentiation capacity [31]. The same authors also reported that all tested sites expressed high levels of MSC surface markers and demonstrated adipogenic and osteogenic differentiation potential [31]. Previous studies revealed differences between bone marrow and adipose tissue-derived pMSC, showing that the former possesses different characteristics; (i) a higher proliferative potential [32], (ii) a proliferation rate that reaching up to 20 passages without modifications in the expression of reprogramming transcriptional factors (Oct4, Sox2, c-Myc, and Nanog) and (iii) ability of differentiation into adipogenic and osteogenic cell lines [32]. Interesting data extending the possible tissue sources of pMSC come from the uterus, where a population of cells able to differentiate in vitro into adipogenic and osteogenic lineages was identified [33]. Those cells expressed MSC markers (CD29, CD44, CD144, CD105, and CD140b) by RT-PCR [33].

Recently, new and interesting results have been obtained by studies on secretome by porcine vascular wall–mesenchymal stem cells (pVW-MSCs), demonstrating a particular composition with high levels of IL-8, GM-CSF, IFN-γ and other immunomodulatory proteins: IL-6 IL-18 IL-4 IL-2 IL-10. Moreover, conditioned medium from unstimulated pVW-MSCs promoted in vitro endothelial angiogenesis, which manifested more strongly when the conditioned medium was from LPS [34].

MSC isolated from some tissues of equine species showed good regenerative potential of soft tissues. This ability seems principally related to the angiogenesis stimulation by trophic factors secreted by equine MSC. In particular, studies revealed that VEGF plays a particular role through its paracrine bioactive factors IL-8, PDGF-AA, ET-1 and IGFBP2 [15] (Table 1). In particular, this last factor isolated for the first time in equine but not in hMSC arouses a great deal of interest for its possible involvement in reparative processes.

Recent innovative studies have been focused to apply new strategies for mapping the equine MSCs surface proteome by using biotin-enrichment and mass spectrometry (MS) analysis in consideration of limited availability of suitable antibodies of high quality. Results demonstrated for the first time in horse species the possibility to use this method and thereby identify 1239 proteins including 19 CD markers.

In particular, regarding chondrogenic differentiation, [35] demonstrated the high efficacy of MSC derived from the synovial membrane (MSC_M_) encapsulated in a three-dimensional alginate hydrogel scaffold differentiated into chondrocytes, opening new possibilities for clinical application in the treatment of joint injuries in horses [36]. Moreover, results obtained from equine umbilical cord blood-derived MSC demonstrating their high proliferative and differentiation capacity towards osteoblasts and chondrocytes, showing a wide possibility of utilization for cartilage tissue engineering and thus offering an indication of a possible transposition of these therapeutic strategies to human preclinical studies [37]. In addition, Arévalo-Turrubiarte et al., demonstrated that synovial fluid-derived MSC, because of their high capacity of proliferation, represent a good source for obtaining MSC in a shorter time suggesting their utilization in therapy [38]. This was in agreement with Gugjoo et al., illustrating the benefits of equine MSCs in inflammation modulation and in healing promotion [27].

A recent innovative method utilizing electroacupuncture (EA) has been tested for mobilization of horse MSC into peripheral blood. The obtained results demonstrated a good efficacy, and the immunomodulatory properties of MSC indicate this method as a valid alternative to surgical procedure [39].

In the last five years a growing interest on feline species, confirmed by a discrete amount of studies, had emerged. Albeit the isolation and the characterization of feline BM-derived MSC started in 2002 [40], several studies evidenced the possibility to isolate MSC from other tissue sources in order to test these cells for treating a range of disorders. Interesting data show that feline Ad-MSC isolated from a young subject are more proliferative in the initial phase of culture than those obtained by a geriatric cat [41]. Differently, when cells are expanded, both cell types were equivalent in respect to their ability to functionally suppress T-cell activation and proliferation [41]. However, it was demonstrated that excess passaging influences some biological cell properties, decreasing both cell proliferation and expression of hematopoietic marker CD45 [42].

The growing interest in veterinary MSC research is a mandate not only to reinforce our current knowledge of the biological properties of MSC for translational human applications but for creation of novel methodologies and therapeutic applications in veterinary medicine too. Indeed, the last decade had experienced a vast majority of animal MSC research investigating new insights into untraditional clinical therapy benefiting human and animal health (Table 2).

In addition, the possibility of in vivo cross-species administration of MSC in a variety of experimental models has been proven in these last ten years. Results from literature show in vivo cross-species administration of MSC in a variety of experimental models. In particular, among the tested animal species, pig MSC showed a good function in a different species, e.g., humans (Li et al., 2012). Moreover, results from a recent paper by Daems et co-workers [43] aimed to use equine MSC for the treatment of osteoarthritis (OA) in dogs, showing that MSC injection was able to reduce pain and lameness respect to the placebo treatment without evidencing adverse events during this study. These findings suggest better investigative mechanisms of action of equine MSCs in the course of OA in future new therapeutic protocols.

Albeit the benefits, limitations in the use of MSC from animal species still exist, some of which are: (i) high variability in differentiation ability and in surface marker expression, (ii) controversy regarding the safety procedures in xenogenic MSC transplantation, (iii) lack of adequate knowledge on the appropriate MSC source to be utilized to cure certain pathologies, (iv) dilemma of ethical concerns are currently being discussed that require the engagement of specialists outside the scientific community. Despite the advancement in the animal field of MSC, further investigations are needed to confirm the effective efficacy of animal MSC in regenerative medicine.

## 3. MSC-Dependent Immunomodulation and Interaction with the Vicinity: Autocrine, Paracrine and Remote Effects

MSC have attracted much attention for their ability to regulate the immune system in vivo and in vitro. Their therapeutic potential is currently being investigated in various immunological disorders such as Crohn’s disease [54], graft-versus-host disease [55], multiple sclerosis [56] and in allergic disorders [57]. The number of clinical trials utilizing MSC therapy continues to increase. Despite widespread preclinical success and their confirmed immunomodulatory effects, MSC-based therapy has not yet reached definite approved clinical therapeutic protocols in autoinflammatory or autoimmune diseases [58]. On the contrary, several MSC products are currently approved for degenerative diseases as arthritis [59] as well as a regenerative tool in anal fistula in certain countries as Canada [60]. This is possibly due, at least in part, to the incomplete understanding of the mechanisms of MSC-based immunomodulation. In the following section, the immunomodulatory potential of MSC and some of the obstacles that are currently hindering defined therapeutic MSC protocols will be briefly discussed. Formerly, MSC were described to have immunosuppressive capacity, since they proved to suppress not only T cell-mediated immune responses by inhibiting T cell proliferation, cytotoxicity and cytokine production, but also the natural limb of the immune system including NK cells [61] and monocytes. MSC are capable of suppressing the differentiation of CD14^+^ CD1a^−^ precursors into dermal/interstitial DC without affecting the generation of CD1a^+^ Langerhans cells through PGE2, IL-10 and downstream signaling via the JAK-STAT pathway [62]. TNF-α-stimulating gene-6 (TSG-6) secreted by MSC has been demonstrated to suppress MAPK and NF-kB signaling activation during the maturation of immature DC into mature DC induced by LPS [63]. MSC have been shown to polarize macrophages from an inflammatory M1 phenotype into an anti-inflammatory M2 phenotype via glucocorticoid receptor and progesterone receptor. In addition, MSC regulate the function of T-reg and increase T-reg chemotaxis [64]. However, studies over the past years have further clarified that when exposed to an inflammatory environment, MSC can orchestrate local and systemic innate and adaptive immune responses as they possess toll-like receptors and the aryl-hydrocarbon receptor [65,66]. Indeed, when present in a stimulatory microenvironment, MSC can stimulate some effectors of the immune system. Several mechanisms have been claimed by which MSC exert their therapeutic immunomodulatory effects. This includes, but is not restricted to, cell-to-cell contact or the release of various mediators, including immunosuppressive molecules, growth factors, exosomes, secretomes, chemokines, complement components and various other metabolites [67]. Bioactive cargo in extracellular vesicles (EV) including proteins, microRNA, and mRNA species can impact signaling responses in target cells to modify inflammatory and repair responses. Starting material, culture conditions and isolation methods appear to impact EV content and potency as demonstrated by a changes in cytokine packaging [68]. The microenvironmental condition where MSC lie greatly influences their mechanism of action and thus their global molecular output. MSC can suppress or activate different members of the immune system depending on the type and the intensity of stimuli received from the microenvironment. Because the immunomodulatory capabilities of MSC are not constitutive but are determined by inflammatory cytokines, the net outcomes of MSC activation might vary depending on the levels and the types of inflammation within the residing tissues. Interestingly, current evidence suggests that MSC exert variable immunomodulatory effects on immune cells depending on the local microenvironment or disease status, thus following the signals they are receiving from the environmental milieu where they reside. LPS-activated MSC have been shown to augment the antimicrobial effects of neutrophils by releasing IL-8 and macrophage migration inhibitory factor (MIF) while suppressing unstrained neutrophil activation via increased production of superoxide dismutase (SOD3), thus attenuating neutrophil-mediated tissue damage [69]. MSC decrease Th1 response in patients with acute graft *versus* host disease (GvHD) and autoimmune diseases such as systemic lupus erythematosus [65]. However, BM-MSC lead to a shift from Th2 to Th1 responses in the airway during allergic inflammatory diseases, including allergic rhinitis and asthma [57]. Inflammatory conditions also have been proven to change immunomodulatory gene expression in MSC or promote the cell-cell contact effect, resulting in an enhanced immunosuppressive response. These observations suggest that MSC are capable of switching their effects to protect the body from disease in different situations. This special phenomenon increased interests in MSC therapy and had encouraged the approval of several clinical trials. However, from another prospective, it increased the challenges MSC are facing for the clinical translation into defined therapeutic protocols due to the diversity of its actions in the presence of a highly variable microenvironment. MSC have long been reported to be immune privileged; this property is thought to enable MSC infusion across major histocompatibility barriers and the creation of off-the-shelf MSC therapies expanded in culture. However, antibodies against MSC and cell-mediated immune rejection of allogeneic donor MSC have been described and suggest that MSC may not actually be immune privileged [70]. Whether rejection of donor MSC influences the efficacy of allogeneic MSC therapies is not known, and no definitive clinical advantage of autologous over allogeneic MSC has been demonstrated [71]. MSC exert therapeutic function through a brief “hit and run” mechanism, (mainly through paracrine effects), therefore protecting MSC from immunodetection. Prolonging MSC persistence in vivo may improve clinical outcomes and prevent patient sensitization towards donor antigens. A recent study had explained some of the controversies as the authors demonstrated that exposure to hypoxia leads to dissociation of 19S and 20S subunits and inactivation of 26S proteasome which prevents degradation of MHC-II and, as a result, MSC become immunogenic. It was concluded that hypoxia-induced inactivation of 26S proteasome assembly instigates loss of immunoprivilege of allogeneic mesenchymal stem cells while maintaining 26S proteasome activity in mesenchymal stem cells preserves their immunoprivilege [72].

In the majority of the completed clinical trials, recipients of MSC-based therapy demonstrated good tolerance and improved clinical symptoms. There remain challenges to the future development of MSC for immunomodulation and a need for improved quality control. Another limiting factor is that MSC for basic research and clinical applications are manufactured and developed as unique cell products by many different laboratories, often under different conditioned media. Immune modulatory effects of MSC are indeed altered by the different expansion media [73]. Human platelet lysate may modulate the immunosuppressive effects of MSC as well as conditioned media. The lack of standardization of MSC properties has limited consensus around which MSC properties are relevant for specific outcomes. The choice of media, cell source, culture environment and storage affect the phenotype and clinical utility of MSC-based products. There are different techniques to prime MSC with specific phenotypes of interest and there is a need for the continued development of standardized assays that provide clinical-grade MSC [74]. Bioequivalence between cell products and batches must be carefully investigated, so that the diversity of phenotypes between different MSC products can be accounted for to identify products with the highest therapeutic potential and to preserve their safety in clinical treatments.

## 4. Challenges Facing Angiogenesis, Bone Healing/Regeneration and other Regenerative Prospectives

MSC enhance angiogenesis by phenotypically switching into the endothelial lineage and mainly exerting a paracrine action into the microenvironment [75]. This is a unique and intrinsic property of all MSC regardless their tissue origin [76], although tissue source influences the stromal secretome [77]. Administration of MSC after a vascular insult enhances a functional revascularization, associated with the upregulation of CD31 [78,79,80], the activation of anti-apoptotic and pro-survival molecular pathways (caspase-3, Bcl-2, Bcl-xL, Akt), a parallel production of a wide range of soluble mediators (VEGF, NGF, HGF, bFGF, IL-6, IL-8, IL-10), and MSC immunosuppressive properties by secreting HLA-G5 [81,82,83,84,85,86,87,88]. In Vitro experiments have confirmed these results, as crossroads of main survival signaling pathways, like ERK1/2, BDNF, CREB or MAP kinases, are targeted and activated [86,88]. Nevertheless, the angiogenic effect is not granted by the sole soluble mediators, rather depending on oxygen level and crosstalk with an often hostile tissue microenvironment, limiting the metabolism of MSC and determining their poor viability after transplantation [89], which still represents an unresolved limitation to the success of many MSC-based clinical trials.

The angiogenic fate of MSC can be also addressed by the degree of hypoxia levels and injury, the in-situ production of inflammatory molecules, the mechanical properties of the tissue [90], the host immune response, and recruiting mechanisms of specific soluble mediators regulating stem cell trafficking and engraftment (i.e., SDF-1 [91,92], EGF or IL-10) resulting in improved survival of surrounding cells [93,94,95]. Additionally, stromal cells can secrete MMPs (responsible for remodeling the extracellular matrix (ECM) components) and respond to stiffness of the tissue, therefore changing their own secretome profile and proangiogenic signaling [90,96]. Besides, ECM can store paracrine factors, acting as a reservoir of molecules regulating angiogenesis [97]. So far, a unique paracrine profile for all MSC types has not been identified, given the strict influence of tissue sources and in vitro culture techniques [98,99], which hamper to finely decode types, amount and functional threshold of soluble mediators by which angiogenesis can be enhanced.

Additional and major drawbacks also originate from the need to define the stromal population by a panel of multiple markers excluding CD31, CD117 and CD34 [1,77,84,85,91,100,101]. In fact, current isolation techniques cannot assure a “pure” stromal and non-endothelial MSC pool. Thus, a minimal residual contamination of endothelial markers could be detected after isolation and propagated through passages. Whether or not the putative endothelial transdifferentiation of MSC once in the tissue arises from the initial and not eradicable fraction of CD31 or CD34 or from a true and direct commitment of the stromal cells is yet to be clarified. The stimulation of the CD34^+^/CD31^−^ MSC pool with VEGF/IGF, enhances endothelial differentiation, strengthening the importance of the endothelial contamination originally present in the stromal fraction [102].

A top priority should be to verify if MSC-derived new formed vessels are only temporarily functioning or they can foster the engraftment of circulating endothelial progenitor and display a specific endothelial metabolic asset including nitric oxide production, response to shear stress and uptake of LDL [91,103]. Researchers are challenged to predict and eventually drive the full restoration of MSC-mediated neoangiogenesis especially after vascular insults, where major shortcomings are based on the low engraftment of transplanted MSC in the tissue, although this event might represent an ancillary mechanism.

Theoretically, the precursors of MSC are the pericytes exerting a physiological and protective role on the vasculature [104]. The supporting characteristic of pericytes resembles the most relevant distinctive mesenchymal-like trait, which combined with the anatomical proximity of MSC to endothelial layers [105], does not fully exclude an even more direct involvement in blood vasculature development. Several studies confirm the role of MSC in stabilizing the structures of neovessels in vivo [105,106]. Accordingly, several molecular pathways have been described. The activation of WNT4 in MSC is demonstrated to enhance blood flow as well as the Wnt modulator Frizzled-related protein-1 improves angiogenesis, highlighting that specific molecular target are responsible of the engraftnent of MSC into vasculature [107,108]. Although reported, the spontaneous transdifferentiation of the stromal progenitor pool into the endothelial lineage represents a rare event [109,110,111,112,113,114], often mixed with the differentiation of MSC into non-endothelial phenotypes [115] and only if induced under certain experimental conditions including standard cocktails of cytokines [116] or combined with shear force, extracellular matrix or 3D scaffolds [117,118,119,120,121]. Interestingly, the in vitro pre-endothelial induction does not improve the performance of MSC in vivo [122], suggesting that an efficient angiogenic reprogramming of MSC results from the mutual biological and molecular inputs between tissue microenvironment and MSC response. Other studies support the paracrine contribution to endothelial cells rather than a direct differentiation into the endothelial lineage. The coculture of endothelial cells and MSC can induce MSC differentiation into smooth muscle cells through the Notch pathway [123], likely activated upstream by TGF-β [124]. TGF-β would also contribute to MSC proliferation and endothelial differentiation through the SMAD–mediated pathways (SMAD 3 and SMAD 2/3, respectively) [125,126]. When Notch is activated in MSC, osteogenesis and angiogenesis can be matched efficiently [127].

We should also consider that the stromal tissue origin determines several biological properties of MSC [77,128], including either a dissimilar balance and expression of pro- and suppressive-angiogenic factors (HGF, IL-10, TGF-β1, INF-γ, trombospondin-1) [129,130] and variation of endothelial cell differentiation and angiogenic potential according to donors [131]. For instance, Ad-MSC are more efficient to sustain vascularisation than the stromal counterpart obtained from umbilical and endometrial tissues [130]. Adult MSC have been never described to contribute to vasculogenesis [132], unless MSC are isolated from a more immature source such as the foetal heart [109], the human amniotic fluid [133], the umbilical cord blood [134], or by cooperating with endothelial and myeloid cells [135,136,137]. These studies reinforce the concept that the restoration of the vasculature can be effectively achieved when the early maturation state of MSC resembles their primitive origin as pericytes or facilitates the endothelial commitment according to tissue microenvironment and to its mechanical properties. In fact, vascular grafts employing MSC on nanofibrous or decellularized scaffolds result in improved reconstruction of the vessels [138,139].

One of the most significant modalities of the MSC-mediated paracrine mechanism is in the form of microvescicles and exosomes, known to control the physiological process of angiogenesis [110,140,141,142,143,144]. The MSC-derived exosomal cargo mainly includes a wide array of specific regenerative and angiogenic microRNAs (miRNAs), targeting gene expression, specific cells and pathways (Wnt pathway, pro-fibrotic signalling mediated by TGF-β and PDGF, collagen production, migration, apoptosis and cell proliferation) within the tissue [145] and acting as mediators of information [146] even when generated de novo by MSC. Notably examples are miR210, 320, 132, 21a-5p and 126 (angiomiRNAs) with a role in vascular repair, angiogenesis and cardio protection [98,147,148,149,150] and therefore strengthening their key role in tissue regeneration.

To date, the precise contribution of some miRNAs derived from extracellular vesicles of stromal origin has been individually established as reported for stem cells which are protected by exosomes-derived MSC through miR22 and Mecp2 pathways upon ischemia [151]. miR675-5p improves osteoblastic differentiation of MSC by activation in the hypoxia pathway and the direct involvement of HIF-1α and Wnt-b signaling [152]. However, a single miRNA could not exactly reflect the effect of the whole cargo in recipient cells.

The MSC-derived miRNA cargo is the product of a balanced mix of positive and negative endothelial modulators, representing an interesting tool to control or even to arrest angiogenesis. In fact, in rheumatoid arthritis, the delivery of the negative modulator miR150-5p by means of exosomes allows direct downregulation of MMP14 and VEGF, both mainly responsible for angiogenic/inflammatory-based clinical complications [153]. Besides, microvesicles do not elicit immunosuppressive responses, boosting the ability of MSC to escape the immune surveillance [77,84,85]. MSC-derived miRNAs can exert angiogenic functions by physical contact, established between MSC and endothelial cells in in vitro coculture systems. The formation of gap junctions allows the transferring of miR200b to the endothelium, resulting in reduced angiogenesis by targeting VEGF, ZEB2, KDR and GATA2 and in increased osteogenic differentiation of stromal cells [154], suggesting that the type and the transferring of specific miRNAs into the microenvironment influence both tissue and MSC fate. Thus, the biogenesis, the relatively high stability and the content of MSC-derived exosomes can vary according to environment and vascular insult. For instance, in in vivo MSC transplantation after myocardial infarction, miR-21a-5p represents the most abundant mediator to decrease proapoptotic gene products such as PDCD4, PTEN, Peli1 and FasL in recipient cardiac cells [155]. Likewise, the microenvironment can also drive the activity of miRNAs. MSC-derived exosomes are particularly sensitive to hypoxic preconditioning, regulating the VEGF/VEGFR axis in the host tissue [156,157,158] or directly, the VEGF content in the exosomes themselves [141]. To date, a clear understanding of the content, subpopulations, biochemical pathways and biological range of actions of MSC-derived extracellular vesicles is missing.

More recently, a role of exosomes has been evidence in relation to the particular involvement of MSCs in the tumor microenvironment and thus in the cancer development metastasis process. In fact, it has well shown that tumor migratory MSCs similar to cancer stem cells (CSCs) act in promoting tumor growing and progression [159]. In particular, MSCs can respond to numerous signals produced by cancer cells and thus are recruited into tumor microenvironment. In this condition, MSCs are “educated” to have pro-metastatic behavior [160]. In this regard, the characterization of several biological mechanisms associated with tumor development evidenced the possibility of exosomes to act in angiogenesis, tumor chemoresistance and on the other side, to exert opposite effects such as the tumor cell apoptosis. According this new scenario, bidirectional communication exists between MSCs and tumors. In addition, MSC-derived exosomes can be re-programmed by tumor-derived small vesicles called “TEX” to exert intense effects on tumor growth [161]. Recent data demonstrated that exosomes stimulated breast cancer cell proliferation and metastasis by transferring tumor-supportive miRNAs and proteins [162]. In this regard, research study on patients demonstrated the possibility to use exosomes as biomarkers for diagnosis and prognosis of breast cancer [163].

Differently, considering the antitumor activity, MSC-derived exosomes seemed to inhibit prostate cancer via delivery of miR-145 by reducing the activity of Bcl-xL and stimulating apoptosis by acting on the caspase-3/7 pathway [164]. More recently, Rosenberger and colleagues demonstrated that exosomes inhibited angiogenesis and tumor growth of oral squamous cell carcinoma by using a model of hamster buccal pouch carcinoma [165].

Thus, the modulation of the mesenchymal secretome is a current reality. Accordingly, packaged microparticles of soluble mediators released by MSC and coated with stromal membranes have being developed to synthetically mimic the angiogenic paracrine asset of MSC, providing the advantage to control the releasing dose and the stability over the time upon myocardial infarction [166]. Alternatively, ECM in the form of hydrogels and obtained by decellularization of the adipose tissue and subsequently loaded with MSC-derived paracrine factors, are promising in vitro biological scaffolds designed to slowly release soluble mediators and improve angiogenesis [167].

Recently, bioengineering approaches by dosing the amount of soluble angiogenic factors in transfected MSC have being a relevant strategy to empower MSC functionality [168]. Alternative promising methods engage genetic manipulation or pharmacological approaches [169,170], attempting to constrain MSC to timely produce a specific profile of soluble mediators and only under specific pathological circumstances. Additional issues remain to be clarified. For instance, it would be important to verify if a suitable balance of pro- and anti-angiogenic factors, rather than merely a range of secreted mediators, might represent a strategic point of control. Moreover, vascular structures cannot be assumed as homogenous within the body; therefore, we should investigate the MSC-mediated angiogenesis according to the specific vascular system of tissues. Finally, further variables such as nitric oxide, oxidative stress and epigenetic mechanisms, known to severely impact angiogenesis [142,171,172], should be carefully considered.

## 5. MSC Plasticity in Their Interaction with Physical Cues

Mechanobiology of stem cells is today a significant issue. Elucidating the interactions between biomaterials and cells has potential to be used for medical purposes. Indeed, the design of biomaterials to mimic natural scaffolds is a novel perspective for developing more efficient stem cell-based therapies in regenerative medicine. To this aim we need to understand that forces generated by adhesion to synthetic extracellular matrices affect stem cell gene expression profiles and ultimately their phenotype. In addition, a better comprehension of how interaction between stem cells and biomaterials, as well as compression or tension changes affects the cells, is also required.

Cells are heterogeneous gels endowed with highly dynamic behaviour in terms of their elastic properties. In this respect, their response to a biomaterial can be monitored as their tensile elasticity, i.e., the Young’s modulus, which measures in pascal units (Pa) deformation when receiving a physical stress. A high Young’s modulus is consistent with a stiff behaviour, indicating reduced ability to deform. Undifferentiated hMSC display Young’s modulus values in the range of kPa magnitude order [144].

As an example, when administered a soluble cocktail inducing an osteogenic phenotype commitment, hMSC show a pronounced decrease in their elastic modulus [173] Although remaining static, cells may receive tensile, compressive or bending stress by the substrate where they are seeded. In this regard, the mechanical stability of networks of cytoskeletal filaments is instrumental to generate the resilience needed to respond to these external forces [174]. Indeed, the physical properties of a scaffold hosting the cells determine a fast and nonlinear actin network remodeling, allowing cell plasticity when they adapt to microenvironmental conditions [175,176]. Soft substrates shape rounded cells, with a low density cytoskeleton. Rigid substrates increase this density, inducing cell spreading [177,178,179]. As a consequence, manipulating the stiffness of a substrate allows us to control physical conditions that are pivotal for stem cell phenotype decision making. These aspects deserve being included in the design of tissue engineering protocols where stem cells onboard of a scaffold are intended as an advanced therapy medicinal product. Thus, in addition to the use of soluble strategies (i.e., cocktails of growth factors), a further approach to address MSC phenotypes is to employ specific scaffold-dependent mechanical cues. It is worthy noting that this aspect has to be controlled also when the objective is to maintain undifferentiated stem cells in culture. In this case, it is crucial to avoid any physical trigger able to prime the cells. In this respect, attention has to be also paid to local surface topography-mediated scaffold cues that have been identified as distinct signals inducing phenotype determination [179,180].

All the above-mentioned mechanical stimuli are in fact activating surface receptors inducing intracellular signaling cascades driving specific molecular activation, such as RhoA and Rho-associated protein kinase (Rock) signaling [181], and concurrent specific and/or gene transcription [182,183,184,185]. In this respect, when multiple clusters of cell integrines firmly bind to the extracellular matrix (ECM), which is the case with stiff substrates, they trigger increased levels of phosphorylated myosin upregulating cytoskeleton tension [186]. Cytoskeletal stress is transferred over nuclear lamin A, which in turn upregulates specific gene expression patterns [187].

Active physiologic mechanical solicitations can be administered as differentiation cue via actuators—namely, bioreactor systems—that are used in vitro to prime cell-based 3D tissue constructs, potentially aiming at a following implant in vivo. Several configurations were designed for different kind of stimuli. Most diffused bioreactor systems are rotating wall systems, spinner flask systems, perfusion system, compression and strain systems [188]. Nowadays, different studies have demonstrated that a “dynamic culture” enriched with proper mechanical stimulation may promote efficient MSC expansion and differentiation in vitro [189,190,191,192,193,194].

## 6. Challenges in MSC Translation into Clinical Practice: The Bone Disease Framework

Other interesting aspects and new perspectives in the field of tissue regeneration and applicative use of MSC are strictly related to their potential to tackle the increase in bone diseases related with the extended life expectancy. Indeed, in many countries bone diseases are becoming a relevant cause of disability, even if they are acute, such as fractures, or chronic, such as osteoporosis and tumours. In any case these pathologies require treatments to enhance the healing and regenerative capacity of bone tissue. The common therapeutic strategies based on bone grafting (autografts or allografts) show some disadvantages. Autografts are limited by the bone volume that can be harvested from the iliac crest and present surgical risks such as bleeding, inflammation, infection, chronic pain, damage at the donor site and morbidity. Allografts also have some limitations, such as the lack of donors, high costs, the need for sterilization and the risk of infectious agent transmission or immune mediated tissue rejection.

These limitations and disadvantages associated with auto- and allograft approaches indicate a clinical need for alternative therapeutic strategies aimed at bone healing and regeneration. Thus, new biomaterials and scaffolds, in association with MSC and growth factors, having requirements of biocompatibility, osteoinductive and osteoconductive properties, are investigated to improve bone repair [195,196,197]. The use of MSC is attractive since they can be harvested from the host with minimal morbidity, they can be modified to secrete osteoinductive factors and implanted on an osteoconductive scaffold, to obtain the three key components leading to osteogenesis [198].

hMSC have been found in several adult tissues, including the synovial membrane, the adipose tissue, the dental pulp tissue, or in perinatal tissues (umbilical cord blood and umbilical cord tissue). All these cells are suited to therapeutic applications for bone regeneration, by means of two different ways of autologous cells transplantation: (1) cell therapies without expansion in culture (one-step procedure) and (2) cell therapies with ex vivo expansion. In the first case, cells are harvested during surgery. In 2010, Jagër et al., successfully treated more than 100 patients with local bone healing disorders using a biomaterial composite in association with BM aspiration concentrate [199]. They observed that the use of BM concentrate reduced the harvest of autogenous bone by 50% without negative effects on bone healing. The second clinical application of MSC includes the autologous cell transplantation after ex-vivo expansion. In 2007, Nöth et al., used a cell population from BM aspirate cultured over 12 days under GMP conditions and transplanted them autologously with a tricalcium phosphate biomaterial for the treatment of femoral head necrosis [200]. The main limitations for clinical application are the sterility technique, long culture time, high cost, and the mixture of human cell culture medium with fetal bovine serum. Thus, recently, orthopedic researchers focused their attention on the clinical use of BM aspirate (BMA) and BM concentrate (BMC) for musculoskeletal regeneration with a “one-step” procedure, avoiding the need of additional laboratory stages and GMP facility, thus reducing costs and risks.

Bone marrow contains MSC, hematopoietic stem cells, endothelial progenitor cells, other progenitor cells and growth factors, including bone morphogenetic proteins (BMP), platelet-derived growth factor (PDGF), transforming growth factor-β (TGF-β), vascular endothelial growth factor (VEGF), interleukin-8 (IL-8), and IL-1 receptor antagonist. It has been identified as an excellent source of cells and growth factors and it has been used with success for bone, cartilage and soft tissue healing [201,202,203].

We focused our attention on the use of BM-derived MSC in spinal fusion surgical procedures, which is the most definitive treatment performed to restore the structural stability of the spinal column in degenerative and oncological spine diseases. Currently, the gold standard stimulus to achieve spinal fusion is autologous bone (autograft), which is commonly harvested from the iliac crest or obtained from the surgical site (local bone). As autologous bone possesses osteogenic, osteoinductive and osteoconductive abilities; the success rate of spinal fusion with autograft is high (up to 95%). However, autograft material is limited in quantity and its quality varies depending on the patient. Moreover, significant morbidity is associated with bone harvesting, as previously described. Between many alternatives proposed to achieve spinal fusion, MSC are interesting because they can provide osteogenic properties allowing bone regeneration.

We recently published a descriptive systematic literature review in order to understand if the use of MSC may represent a valid strategy to facilitate and accelerate bone regeneration and fusion during spine surgery [204]. In this review we selected and analyzed 50 relevant papers, stratified according to preclinical studies and clinical trials. Preclinical published data showed that MSC have the potential to achieve, facilitate and accelerate spinal fusion. However, preclinical studies on animal models indicated that some barriers remain prior to this therapy translation into the clinical application.

So far, few published clinical studies employ stem cells for spinal fusion [204,205]. Most of them used concentrated autologous BM as primary source of stem cells and they showed fusion results ranging from 87% to 92.3% in a total of 297 patients [206,207,208,209,210,211,212,213,214].

Despite these results, some critical limitations exist, including the site of BM harvest, the choice of the optimal cell concentration, the methods to obtain the maximum number of cells, the delivery method (appropriate scaffold), the ideal manipulation procedure (one-step or ex vivo expansion procedure) and the best implantation technique. Thus, more in vitro and animal studies are useful to highlight these concerns and randomized controlled clinical trials are necessary to carefully evaluate the safety and efficacy of MSC use in spine surgery.

In this scenario, our in vitro studies focused on the site of BM harvest, as the withdrawal from iliac crest during spine surgery leads to an increase in operative and rehabilitation time and to possible donor site morbidity. We previously observed that stromal populations from different sources, although immunophenotypically similar, display distinct signatures of HOX and TALE homeobox genes [215]. Our data suggested that cell populations derived from different body sites may represent equivalent cell sources for cell-based therapeutic strategies for regeneration and repair of specific tissues. In light of these observations, we analyzed and compared the in vitro proliferation and differentiation activities of MSC derived from different body sites (iliac crest, sternum and vertebrae, colon and dental pulp). Our results demonstrated that MSC derived from vertebrae (vMSC) generated mature cells of all mesenchymal lineages (osteocytes, adipocytes and chondrocytes) following exposure to specific inducing agents, with greater efficiency than MSC derived from other sources [216]. This finding could be very interesting and open new perspectives for the use of MSC to improve spinal fusion, considering that in the course of a spine surgical procedure vertebral bone marrow can be easily harvested in an amount proportional to the length of the arthrodesis, simultaneously with the preparation of the site for pedicle screw insertion.

Afterwards, we analyzed the strategies developed for cell-based therapies, encompassing in vitro expanded MSC and “one-step” procedures using BM in toto (i.e., BMA) or BMC. Specifically, using BMA appears as a promising technique, showing increased regenerative potential by the addition of marrow elements and the possibility to perform the entire procedure directly in the operating room [217,218].

**Figure 1:**
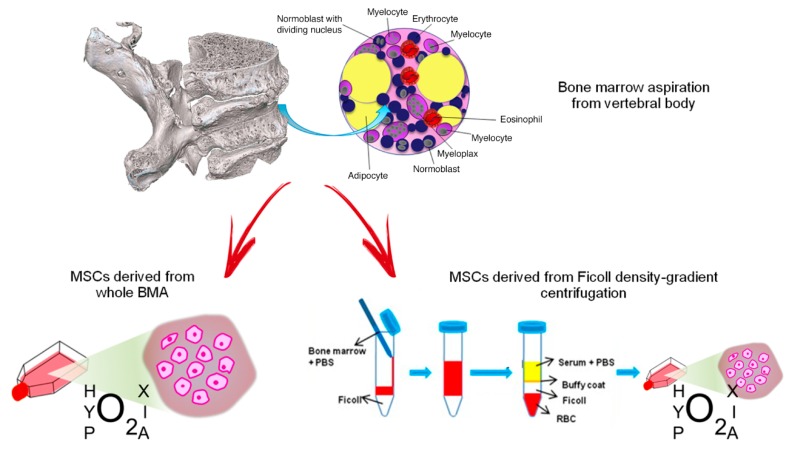
MSCs derived from vertebral bone marrow aspiration: culture of whole BMA or isolation of MSCs from density-gradient centrifugation. Red arrows indicate the two different processes to obtain MSCs from vertebral bone marrow aspirate (BMA): on the left, MSCs derived from whole BMA are cultured in hypoxic conditions; on the right, MSCs are isolated from BMA by Ficoll density-gradient centrifugation and they are cultured in hypoxic conditions. Blue arrows indicate the passages to obtain isolation of MSCs from BMA by Ficoll density-gradient.

Following collection of vertebral BMA samples, we analyzed the biological characteristics and HOX and TALE gene expression in vMSC derived from whole BMA and from density-gradient centrifugation of BMA, both cultured under the hypoxic stimulus, in order to identify the best cell isolation technique and condition for spinal surgery application [219] (Figure 1).

Our results showed that the in vitro expansion rate and CFU potency of vMSC derived from whole BMA under hypoxia were increased compared to all the other culture conditions analyzed.

Although all culture conditions generate mature cells of all mesenchymal lineages when induced under osteogenic, adipogenic and chondrogenic differentiation, RT-PCR showed a significant upregulation of RUNX2 and ALPL in vMSCs derived from whole BMA under hypoxia in comparison to normoxia. A different behavior was observed in vMSCs derived from density gradient centrifugation where a lower expression of COL1A1 was observed in hypoxia in comparison to normoxia. This lower expression of COL1A1 under hypoxia indicated a decreased efficiency in the final osteogenic potential, previously observed [220,221,222] This aspect was particularly evident when vMSCs derived from whole BMA and from density-gradient centrifugation were compared under hypoxia; a significant up-regulation of all genes markers of osteogenic differentiation, i.e., COL1AI, ALPL, RUNX2 and OPG, were seen in vMSCs derived from whole BMA. The higher expression level of these osteogenic markers can prove the superior bone formation ability of vMSCs derived from whole BMA cultured under hypoxia.

In vMSCs derived from whole BMA, it was seen that hypoxia strongly up-regulated the expression of chondrogenic genes SOX9 and ACAN in comparison to normoxia. Similarly, vMSC derived from density gradient centrifugation highlighted an upregulation of ACAN. However, in vMSCs derived from whole BMA, contrary to osteogenic and chondrogenic gene expression, adipogenic genes, PPAR-γ and ADIPOQ, were up-regulated under normoxia. These results indicate that vMSCs derived from whole BMA under hypoxia differentiated onto the adipogenic lineage following exposure to specific inducing agents but with a lower efficiency in comparison with all the other culture methods and condition. Thus, we hypothesized that low oxygen tension in vMSCs derived from whole BMA reduced adipogenesis, as postulated by other authors, in a hypoxia inducible factor (HIF)-1 dependent manner.

This is interesting for the improvement of spinal surgery where the hypoxic condition of the site would facilitate the differentiation of vMSC towards osteoblasts, inhibiting adipogenesis and favoring the initiation of proper bone regeneration [219].

We also addressed the question of whether vMSCs isolated with different techniques might, following osteogenic induction, modulate the expression levels of HOX and/or TALE genes. We found that vMSCs derived from whole BMA up-regulated HOXB8 under hypoxia. HOXB8 is involved in the expansion of hematopoietic stem and early progenitor cells and it is also implicated in vertebral development, since its inactivation causes slight vertebral abnormalities while its increase can help by-pass the block of posterior elongation of axial tissues [223,224,225]. Consequently, HOXB8 could have a key role also in the regulation of adult vMSCs. Additionally, comparing the differences between vMSCs derived from whole BMA and from density-gradient centrifugation under hypoxia, we detected higher HOXB8 expression in vMSCs derived from whole BMA [219].

The results of this study showed for the first time that hypoxic preconditioning of vMSCs derived from whole BMA can enhance proliferation, morphology, osteogenesis and chondrogenesis, inhibit adipogenesis and up-regulate distinct level of HOX signatures. These aspects could open new perspectives for the improvement of spinal surgical procedures wherever the hypoxic nature of the site would facilitate the differentiation of vMSCs towards osteoblasts, giving the biological rationale for the use of vertebral bone marrow in spinal surgery.

Because of the presence of megakaryocytes and platelets, BMA is prone to clotting, even after the addition of anticoagulants [226]. When we collected samples of vertebral bone marrow from the operating room for in vitro analysis, although they were mixed with anticoagulant immediately after withdrawal, many samples were at least in part clotted when they arrived in the laboratory for processing. In order to maximize cell yield, we tried to culture both un-clotted BMA and clotted BMA (mechanically cut) and after 15 days of culture we observed higher growth kinetics of MSC derived from clotted compared to un-clotted BMA [218]. These results suggested that clotted BMA might be a more efficient source of MSC than unclotted BMA and that BMA clot could be entirely transplanted to the lesion site alone or in association with scaffolds. Results reported from preclinical studies are confirming that BMA clot could be able to perform the necessary physiological functions to achieve and facilitate cartilage and bone tissue regeneration in patients [218]. Recently, we also demonstrated the better efficiency of BM-MSC with respect to Ad-MSC in a preclinical study performed on a rat model of spinal fusion, using a strontium substituted β-tricalcium phosphate as a scaffold, associated with unexpanded and undifferentiated MSC [227].

According to our data [216,218,219], vertebral body MSC derived from whole BMA exhibited more suitable biological characteristics for bone regeneration and specific levels of HOX gene activation, providing an alternative source for tissue engineering applications to spinal surgery.

We support the idea that stem cells have a big potential that can be exploited to treat many diseases and structural damages using cell therapy and regenerative medicine strategies. However, as previously observed, some limitations have to be overcome in order to use MSCs in clinical settings [228]. Most concerns are about the in vitro expansion of MSCs and their clinical application. Our challenge is to overcome this issue using a “one-step” procedure, where stem cells harvested from the patient’s vertebral pedicles are used in the same patient during the same surgical procedure.

Moreover, the results of recent studies suggest that MSCs found in various niches of perinatal and adult tissues have a common source, primary mesenchyme. After migration from the mesenchyme during development, they then adapt to the designated niche. MSCs from each of the niches maintain certain common characteristics (i.e., expression of markers) while varying in others (i.e., self-renewal and differentiation potential). This observation suggests that in clinical applications it is necessary to select the correct tissue for deriving MSCs in order to achieve the greatest outcome for the treatment of a particular disease. Following in vitro studies, we propose to use vertebral bone marrow as best source of MSCs for spinal fusion.

In conclusion, based on our previous data, we are setting up a clinical trial in which vertebral bone marrow harvested from pedicles during the surgical procedure is re-implanted in the arthrodesis area through a “one-step” procedure, using an appropriate scaffold, without any additional surgical time or any involvement of other anatomical sites. In our opinion, the results of this trial could add new insights into the potential of MSCs in the clinical reality.

## 7. Conclusions

The worldwide growing interest in MSC and their possible utilization in therapy has evolved to studies aimed to deepen the knowledge about MSC physiology, including issues such as their behaviour in response to autocrine and paracrine factors, their survival time, ability to migrate into organs and tissues and their donor-to-donor variability. All of these aspects certainly could complete the existing framework about MSC biology by providing support in the communication between basic scientists and clinicians for more useful applications in therapy. In fact, with the current review, we have tried to demonstrate data from basic science to clinical trials not only for a substential overview on the current status of human and animal MSC but highlighting the limitations and challenges MSC continue to face despite the advancement of the field.

In the near future, opportunities offered by new synergies and disciplines might stimulate research at the interface between biomedical sciences, engineering and clinical application to enhance the translation of MSC from basic research into the clinics.

The discovery of the possible use of MSC derivatives, such as conditioned medium, exosomes and extracellular vesicle, represents a promising aspect for future clinical applications where active MSC products might act as substitute mediators of local MSC action in regenerative medicine protocols.

## Figures and Tables

**Table 1:** Growth factors and their influence on biological activities of MSC from animal species.

Animal Species	MSC Origin	Growth Factor	Role	Reference
Dog	Bone marrow	PDGF and bFGF	Growth factor and proliferation	[9]
	Adipose tissue	PRGF, FGF-2	Proliferation and chondrogenesis	[11,12]
Horse	Bone marrow	IGF-1	Cell proliferation and collagen and GAG synthesis	[13]
	Adipose tissue	TGFβ3	Tenogenic differentiation of equine MSC	[14]
Pig	Bone marrow	VEGF	Angiogenesis	[15]
Sheep	Bone marrow	EGF + bFGF + TGFβ	Proliferation, migration and invasion	[16]

**Table 2:** Preclinical studies of animal-derived MSC as possible therapeutic potentials.

Animal	N. Subjects	MSC Type	Disease	Treatment	Effects	Ref
Cat	1	Spinal	Spinal cord injury	Autologous MSC (7 × 10^8^) + collagen	Significant functional clinical improvement; long melioration	[44]
	6	AdMSC	Chronic kidney	Allogenic MSC (2 × 10^6^ cells; 2–6 weeks)	Long term melioration	[45]
Dog	130	Micro fragmented AdMSC	Osteoarthritis	Intra-articular injection	Long-term pain control	[46]
Horse	10	BM	Cartilage defects	Intra-articular injection (2 × 10^6^ cells)	Increase in repair tissue firmness	[47]
	33	BM	Femorotibial lesions (meniscal, cartilage or ligamentous)	Intra-articular injection (1.5 × 10^7^–2.0 × 10^7^ cells)	Improvement	[48]
Pig	1	BM	Model of intervertebral degeneration	Autologous (1 × 10^6^ cells/mL)	Reduction of degenerative process	[49]
	8	AdMSC	Esophagus	Cells implanted on scaffold	Regrowth of esophageal tissue	[50]
	2	BM	Cutaneous wound healing	Autologous MSC (1.5 × 10^7^ cells) injected intradermally	Regeneration	[51]
Sheep	10	BM	Osteoarthritis	Autologous MSC injected intra-articular	Improvement of articular cartilage	[52]
	6	PB-MSCs	Cutaneous wound healing	Injection (1 × 10^6^ cells) intradermally	Skin re-epithelialization	[29]
	1	AdMSC and BM	Osteoarthritis	Autologous chondrogenic induced Ad and BM cells	Improvement of articular cartilage within 6 weeks post-treatment	[53]

## Abbreviations

Ad-MSC	adipose-MSC
BDNF	Brain-derived neurotrophic factor
BM	bone marrow
BMA	bone marrow aspirate
BMC	bone marrow concentrate
CFU	colony-forming unit
cMSC	canine MSC
ECM	extracellular matrix
FGF	fibroblast growth factor
HGF	Hepatocyte Growth Factor
HIF-1α	Hypoxia-inducible factor 1-alpha,
INF	Interferon
hMSC	human MSC
IL	Interleukine
MCP-1	Macrophage chemotactic factor 1
MMP-14	Matrix metalloproteinase-14
MSC	mesenchymal stem cells
MSC_M_	mesenchymal stem cells derived from synovial membrane
oMSC	ovine MSC
NGF	Nerve growth factor
PDGF	Platelet derived Growth Factor
pMSC	pig MSC
TGF-β	Transforming Growth Factor Beta
VEGF	Vascular endothelial growth factor
vMSC	Vertebra-derived MSC
